# Diagnosis of a Ruptured Pulmonary Hydatid Cyst in a 26-Week Pregnant Female With Bedside Lung Ultrasound in Emergency (BLUE) Protocol: A Case Report

**DOI:** 10.7759/cureus.25431

**Published:** 2022-05-28

**Authors:** Banu Arslan, Osman Sonmez

**Affiliations:** 1 Department of Emergency Medicine, Basaksehir Cam and Sakura City Hospital, Istanbul, TUR

**Keywords:** lung ultrasound (lus), blue protocol, pregnancy, intrabronchial rupture, pulmonary hydatid cyst

## Abstract

Pulmonary hydatid cyst during pregnancy is extremely rare and life-threatening for the mother and fetus. Throughout pregnancy, hydatid cysts may enlarge due to the suppression of cellular immunity and steroids secreted from the placenta. In late pregnancy, the cysts can reach a huge volume with an increased risk for subsequent rupture due to the compression of the enlarging uterus and anaphylactic shock. Intrabronchial rupture is a rare and life-threatening complication of pulmonary hydatid cysts. It is vital to diagnose it as early as possible and manage patients with surgical intervention with aggressive medical treatment. Plain radiograph, computed tomography (CT) scan, and magnetic resonance imaging (MRI) can be used to identify pulmonary hydatid cysts. However, the diagnosis of hydatid cyst is quite challenging in pregnant patients due to concerns of radiation. Herein, we present a 26-week pregnant patient with acute respiratory failure. Bedside lung ultrasound was notable for thickened and severely broken pleural line with a large subpleural consolidation, and a giant fluid-filled cyst covered almost the entire left thorax, causing a mediastinal shift. In the present case, we highlighted that the bedside lung ultrasound in emergency (BLUE) protocol is an easy, safe, and fast way to identify pulmonary hydatid cyst. It should be the initial technique of choice for the diagnosis of pulmonary hydatid cysts in pregnant patients.

## Introduction

Hydatid disease occurs in the larval stages of taeniid cestodes of the genus *Echinococcus* [1]. The majority of human infestations occur by *Echinococcus granulosus*, which causes hydatid cysts [1]. The most involved organs are the liver (55%-70%) and the lung (18%-35%); however, hydatid cysts can be found in many organs such as the kidney, spleen, skin, and muscles [2]. Hydatid cysts are globally distributed and are concentrated in sheep-raising areas. The incidence of hydatid disease in pregnancy is reported as one in 20,000-30,000 [3]. Pulmonary hydatid cyst during pregnancy is rare and life-threatening for the mother and fetus [4]. The incidence of this rare condition is unknown.

Hydatid cyst grows slowly, and patients may remain asymptomatic for years. Depending on the organs involved and size, the clinical presentation can include a variety of signs and symptoms. Chronic cough, chest pain, shortness of breath, and hemoptysis may present when the lung is affected [5]. However, rare presentations such as anaphylactic shock are also reported [6]. The cyst may be perforated spontaneously or because of trauma. Pulmonary hydatid cyst rupture may associate with acute-onset cough, shortness of breath, and fever. Besides, coughing up clear, salty fluid and hydatid membrane fragments may occur when the cyst evacuates into the airway, as was observed in the present case.

Hydatid cysts may enlarge in pregnancy due to the suppression of cellular immunity and steroids secreted from the placenta. Large cysts can also shift the mediastinum, causing the atelectasis of the adjacent parenchyma, as it is in the present case. In late pregnancy, the cysts can reach a huge volume with an increased risk for subsequent rupture due to the compression of the enlarging uterus and anaphylactic shock [4]. Herein, we present a 26-week pregnant patient with a ruptured pulmonary hydatid cyst.

## Case presentation

A 24-year-old pregnant female at 26 weeks of gestation was transferred to our emergency medicine department with acute-onset shortness of breath. The patient reported three hours of feeling like drowning and coughing up clear fluid that worsened when lying down. History was notable for seven months of a dry cough that gradually worsened in the last two months. She did not report any fever, chest pain, or weight loss. Two weeks ago, she visited her primary care physician and was prescribed antibiotics for pneumonia. However, clinical improvement was not reported. She denied new pets, environmental exposure, or sick contacts including coronavirus. She is a stay-at-home mom and has no medical conditions and no history of smoking, drinking, or recent travel. However, she grew up in Southeastern Anatolia where a hydatid cyst is a common health problem.

On physical examination, the patient was found to be anxious and severely dyspneic. Clinical examination revealed tachycardia and tachypnea, with the use of accessory respiratory muscles and adopting a tripod position. On admission, her temperature was 36.5°C, blood pressure was 112/68 mmHg, heart rate was 135 bpm of regular rhythm, respiratory rate was 35 per minute, and oxygen saturation was 78% on an oxygen mask at 6 L/minute. The other striking examination findings were sweaty, cold, and clammy skin with peripheral cyanosis. Pulmonary auscultation was notable for decreased breathing sounds on the left and wheezes all over the chest. The remainder of the physical examination was normal, including appropriate weight gain for the second trimester of her pregnancy.

**Figure 1:**
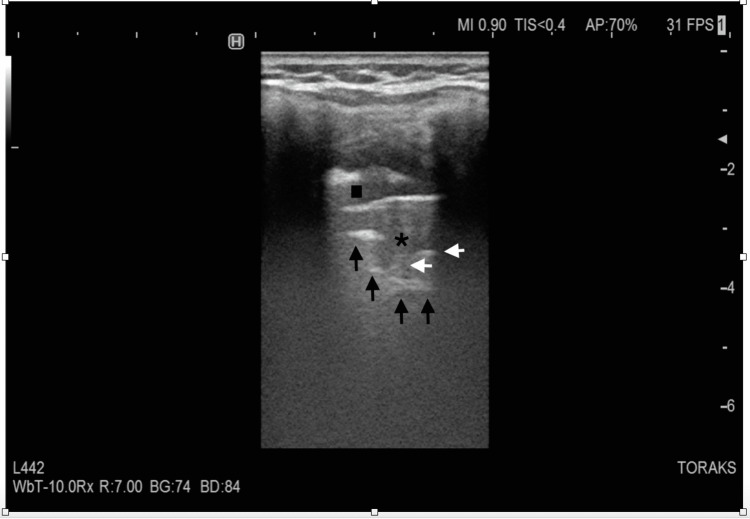
Lung ultrasound of the left upper BLUE point The figure demonstrates no pleural sliding, thickened and severely broken pleural line (square), consolidated areas (asterisks), air bronchograms (white arrows), and the shred sign (black arrows).

**Figure 2:**
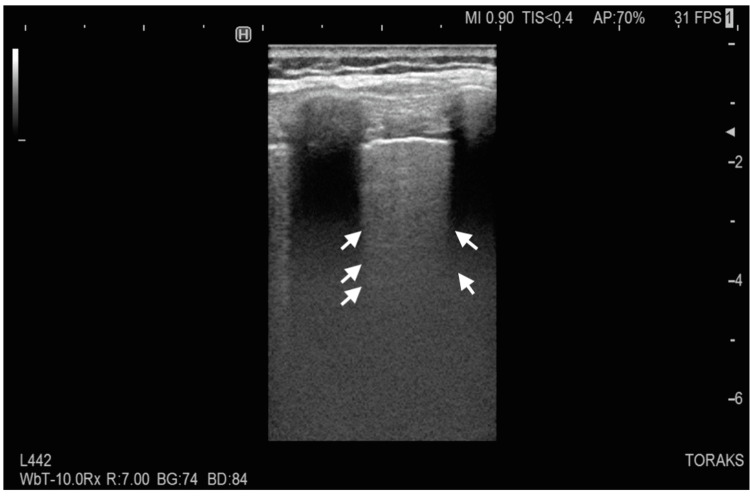
Lung ultrasound of the left lower BLUE point The figure demonstrates no pleural sliding. White arrows indicate areas with coalescent B-lines, called white lung.

**Figure 3:**
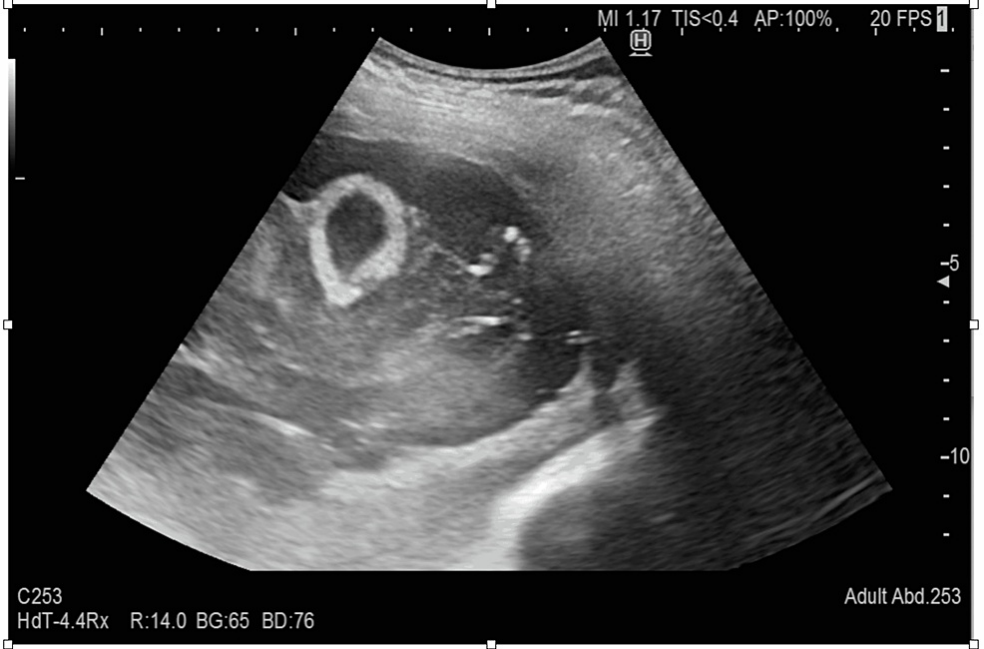
Lung ultrasound of the left lower posterior zone (PLAPS point) The figure demonstrates a complicated cyst.

**Figure 4:**
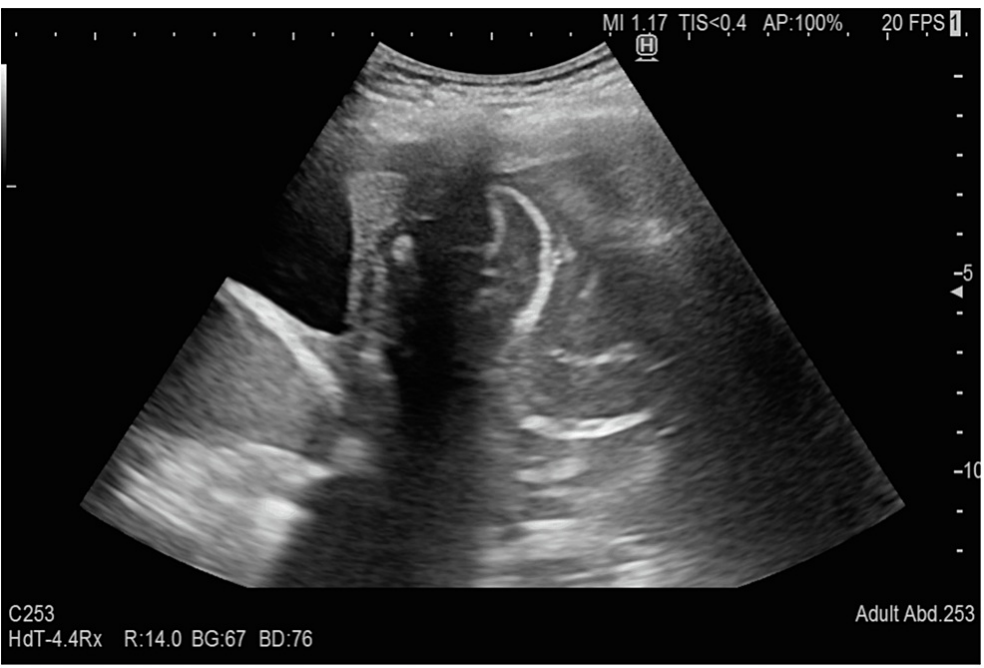
Lung ultrasound of the left lower posterior zone (PLAPS point) The figure demonstrates a complicated cyst.

The emergency medicine physician performed the BLUE protocol to explore acute respiratory failure. A phased array probe was used (Sonosite C-60, 5-2 MHz; Sonosite, Bothell, WA, USA) with a vertical probe orientation. Left upper and lower BLUE point examination was significant for thickened and severely broken pleural line with a large subpleural consolidation (Figures 1, 2). On the left posterolateral alveolar and/or pleural syndrome (PLAPS) point, there was a suspicion of a large fluid-filled cyst that covered almost the entire left thorax (Figures 3, 4). The right lower BLUE point was significant for an area of consolidation. The bedside abdomen ultrasound was unreliable due to limited positioning. An echocardiogram revealed that the tip of the heart was positioned on the right side of the chest. Additionally, the right ventricle, the tricuspid annular plane systolic excursion (TAPSE), and the size and ejection fraction of the LV were demonstrated as normal (65%).

**Figure 5:**
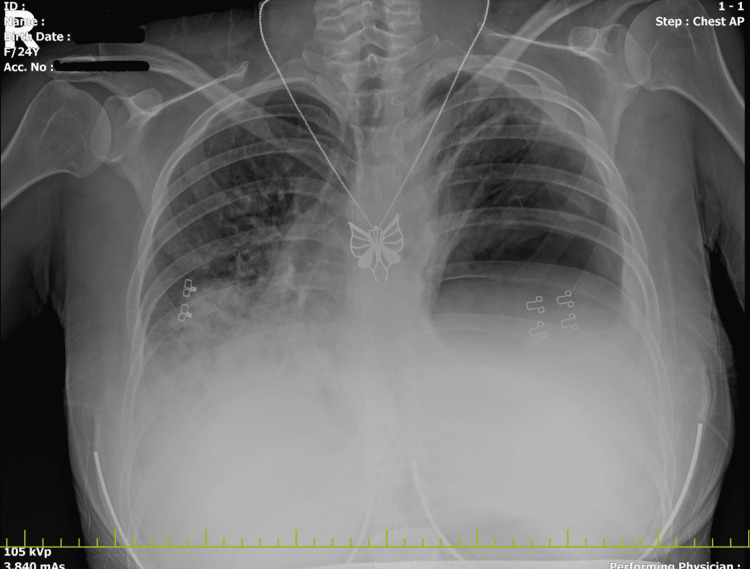
Chest X-ray Huge cystic lesion with an air-fluid level located in the lower and mid-zone of the left lung causing mediastinal shift.

Given the patient’s life-threatening symptoms, an immediate chest X-ray was warranted (Figure 5). The chest X-ray demonstrated a huge cystic lesion with an air-fluid level located in the lower and mid-zone of the left lung. It obscured the left ventricular margin and costophrenic sinus and displaced the mediastinum to the right. Adjacent parenchymal consolidation in the right lung was also documented.

**Figure 6:**
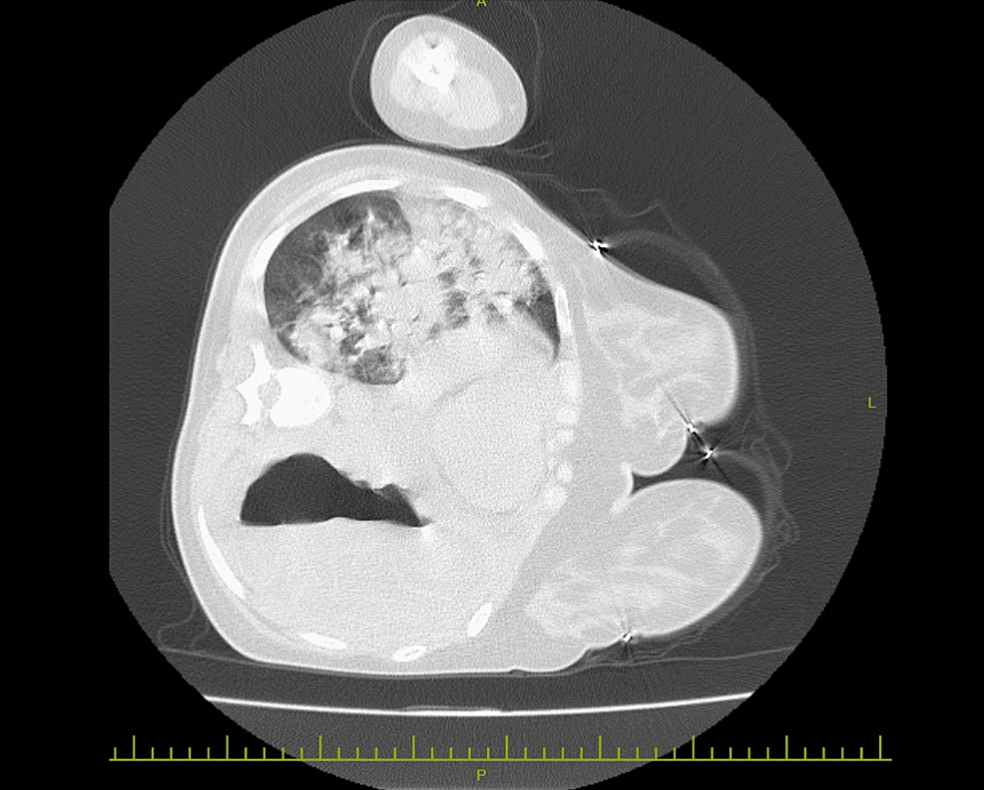
CT scan of the chest (lung window) A giant cystic lesion with an air-fluid level on the left and air bronchogram containing pulmonary consolidation on the right lung.

**Figure 7:**
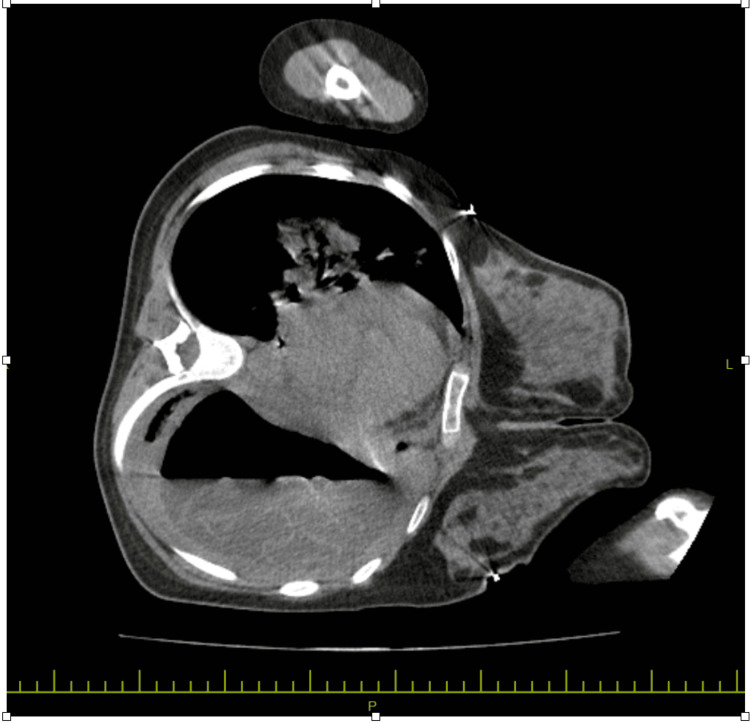
CT scan of the chest (mediastinal window) A giant cyst measuring 11 × 16 cm with a thick wall and floating hydatid membranes in the pleural fluid (serpent sign).

Since the patient’s symptoms including a feeling of drowning and coughing up a clear fluid were provoked by lying back, a computed tomography (CT) scan of the chest was done while the patient was lying in the left lateral decubitus position. A giant cyst measuring 11 × 16 cm with a thick wall and floating hydatid membranes in the pleural fluid (serpent sign) was demonstrated (Figures 6, 7).

Arterial blood gas analysis on admission revealed evidence of type 1 respiratory failure: sO2: 80%, pO2: 46.2 mmHg, PCO2: 34.1 mmHg, pH: 7.41, and HCO3: 21.6. Her complete blood count (CBC) showed anemia with mild neutrophilic leukocytosis. Renal and liver functions were unremarkable. Serologic studies came positive for *Echinococcus granulosus*, confirming the diagnosis of a hydatid cyst.

A non-rebreather facemask (NRFM) at 14 L/minute with salbutamol therapy was immediately started. Intravenous piperacillin/tazobactam 4/0.5 g every eight hours was initiated for pulmonary consolidation located in the lower and mid-zone of the right lung. After careful consideration, oral albendazole at a dose of 2× 400 mg was added to the therapy.

The patient was admitted to the cardiovascular intensive care unit, and an emergency operation was planned. A decision between the intensivist, thoracic surgeon, obstetrician, and neonatologist was made to proceed with the continuation of the pregnancy. During the surgery, VA-ECMO support was initiated to improve oxygenation. The diagnosis of a ruptured hydatid cyst was confirmed by surgery and histopathological examination. The early postoperative period was complicated with intrauterine fetal demise and patient death three days later.

## Discussion

The diagnosis of a pulmonary hydatid cyst during pregnancy might be challenging and delayed due to radiation risk concerns for imaging studies. Ultrasound, plain radiograph, computed tomography (CT) scan, and magnetic resonance imaging (MRI) can be used to identify a pulmonary hydatid cyst. Although it is largely accepted that lung ultrasonography is beneficial to assess peripheral lesions and pleura, we believe that bedside lung ultrasonography should be used as an initial imaging modality in pregnant patients, since it mitigates maternal concerns for radiation. In the present case, we adopted the bedside lung ultrasound in emergency (BLUE) protocol to explore the patient’s acute respiratory failure. Although the BLUE protocol is designed to assess main diseases including pneumonia, congestive heart failure, chronic obstructive pulmonary disease (COPD), asthma, pulmonary embolism, and pneumothorax, we demonstrated that a pulmonary hydatid cyst can also be identified with this technique.

There are three standardized points used in the BLUE protocol. In this technique, the upper hand is placed on the anterior thorax horizontally with the upper little finger just below the clavicle and fingertips at the middle line. The lower hand is placed just below the upper hand. Thumbs are not included. The upper BLUE point is at the middle of the upper hand. The lower BLUE point is at the middle of the lower hand palm. In order to locate the posterolateral alveolar and/or pleural syndrome point (PLAPS) point, a horizontal line from the lower point is drawn until the posterior axillary line is reached [7].

Ultrasonography can be validated by other imaging techniques including plain radiograph, computed tomography (CT) scan, and magnetic resonance imaging (MRI). In most cases, a chest X-ray is the initial imaging modality. The typical findings in chest X-rays are one or more well-defined and rounded homogeneous density mass surrounded by normal lung tissue [8]. On CT scan, uncomplicated pulmonary hydatid cysts appear as well-circumscribed homogeneous lesions with low-density and smooth hyperdense walls of variable thickness. If the cyst is complicated, a variety of radiologic signs can be seen such as the air crescent sign, air bubble sign, Cumbo sign, serpent sing, water lily sign, and rising sun sign [9]. On MRI, cysts show low signal intensity on T1-weighted images and high signal intensity on T2-weighted images [10].

The management of this disease is quite challenging due to the lack of standardized treatment protocols. Surgery is the preferred treatment. Preoperative treatment with albendazole is still controversial; however, it reduces the risk of recurrence [11].

## Conclusions

Although many imaging modalities have been used in the diagnostic approach, we recommend that bedside lung ultrasonography should be the initial technique of choice for the diagnosis of pulmonary hydatid cysts in pregnant patients.
